# Supplementary figures and images for: Anti-CTLA-4 and anti-PD-1 immunotherapies repress tumor progression in preclinical breast and colon model with independent regulatory T cells response
Source: Transl Oncol. 2022 Mar 24;20:101405. doi: 10.1016/j.tranon.2022.101405 (PMC8961218; doi:10.1016/j.tranon.2022.101405)

Figure S1

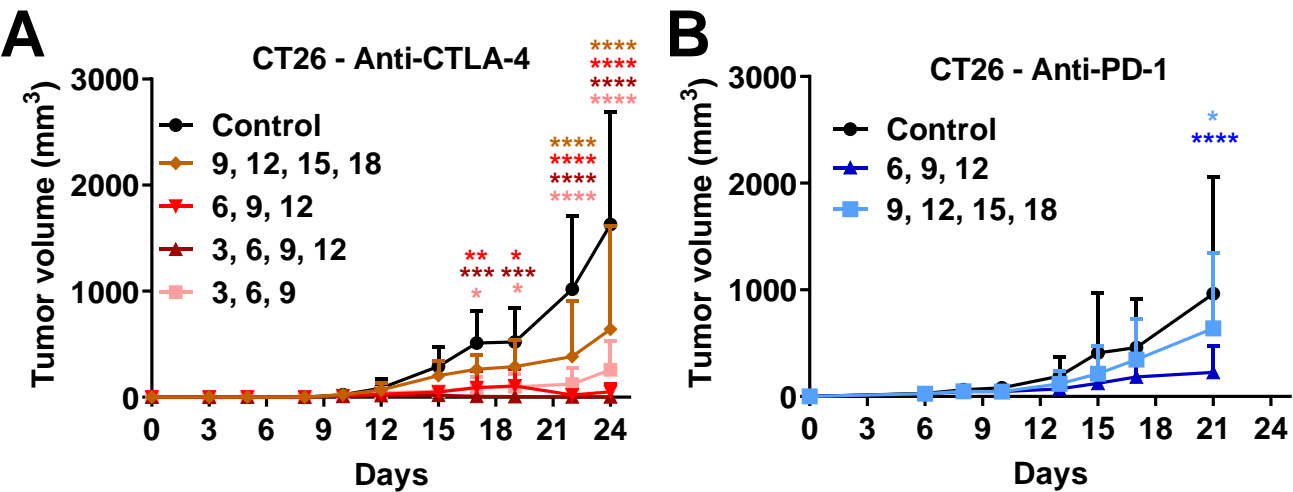

Supplement: Supplementary file 1 [file mmc1.pdf]

Figure S2

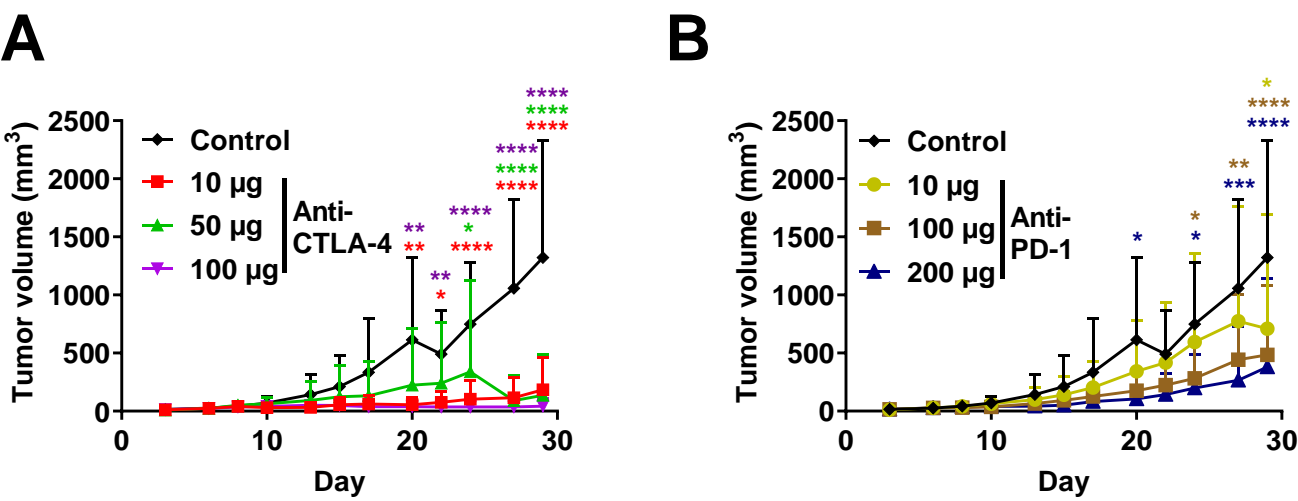

Supplement: Supplementary file 2 [file mmc2.pdf]

Figure S3:

All subtypes  
TNBC

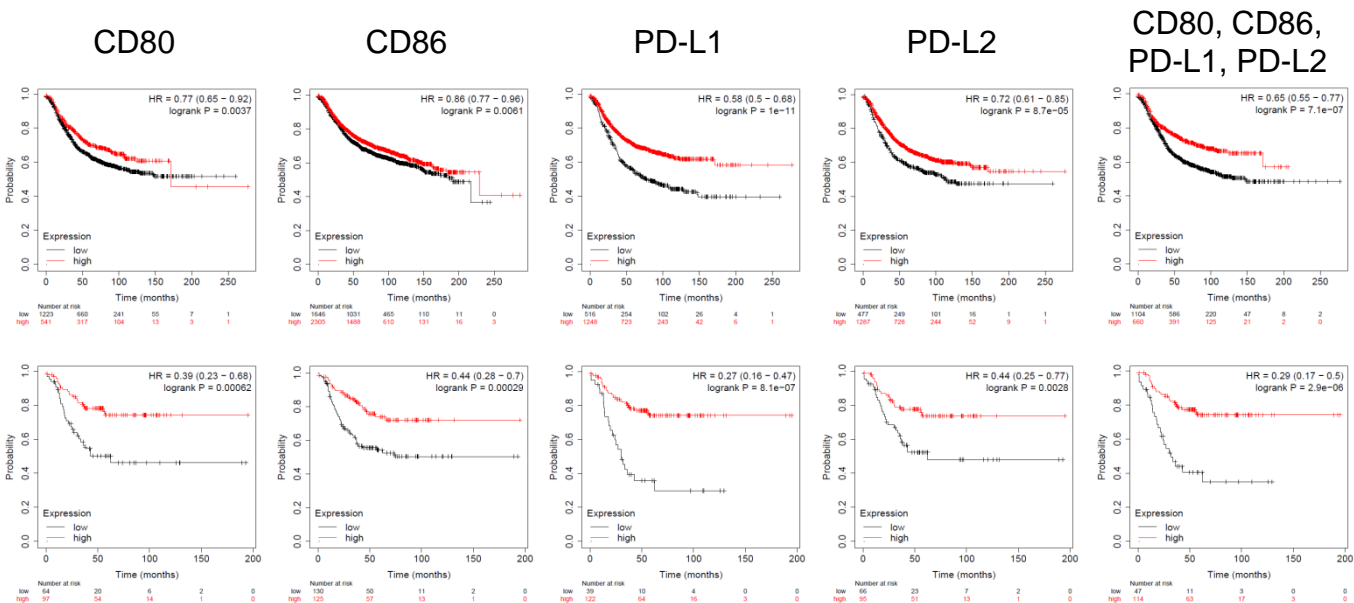

Supplement: Supplementary file 3 [file mmc3.pdf]

Figure S4

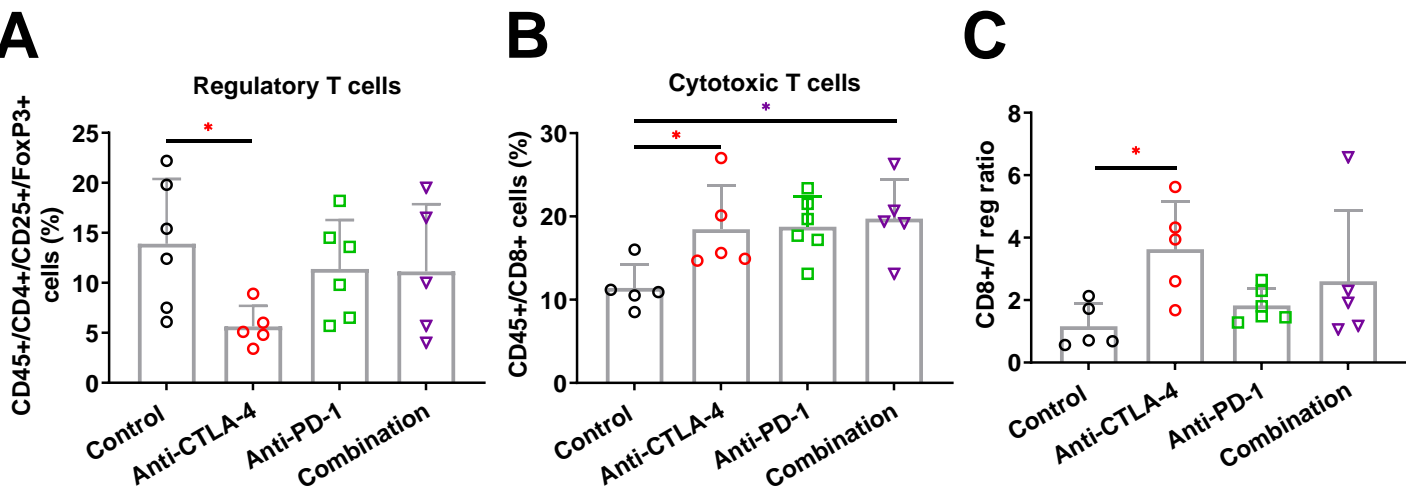

Supplement: Supplementary file 4 [file mmc4.pdf]
