# Supplementary material for: Anti-CTLA-4 and anti-PD-1 immunotherapies repress tumor progression in preclinical breast and colon model with independent regulatory T cells response
Source: Transl Oncol. 2022 Mar 24;20:101405. doi: 10.1016/j.tranon.2022.101405 (PMC8961218; doi:10.1016/j.tranon.2022.101405)
Supplement: Supplementary file 7 [file mmc7.docx]

**Anti-CTLA-4 and anti-PD-1 immunotherapies repress tumor progression through independent cellular mechanisms**

Tristan Rupp^1,#^, Laurie Genest^1^, David Babin^1^, Christophe Legrand^1^, Marion Hunault^1^, Guillaume Froget^1^, Vincent Castagné^1^

Running title: Anti-CTLA-4 and anti-PD-1 repress tumor progression

# corresponding and lead author

[trupp@porsolt.com](mailto:trupp@porsolt.com), [rupptristan@hotmail.fr](mailto:rupptristan@hotmail.fr), Phone: 0033 (0)2 43 69 36 07

^1^ Porsolt SAS, French preclinical contract research organization (CRO), ZA de Glatigné, 53940 Le Genest-Saint-Isle, France

The authors declare no potential conflicts of interest

**Highlights**

- Anti-PD-1 and anti-CTLA-4 induced anti-tumor response in breast cancer mouse model.
- Anti-PD-1 and anti-CTLA-4 induced anti-tumor response in colon cancer mouse model.
- Anti-CTLA-4 reduced colon cancer–derived lung metastasis formation in a mouse model.
- We identified specific T cell response between anti-PD-1 and anti-CTLA-4.
