# Supplementary material for: Anti-CTLA-4 and anti-PD-1 immunotherapies repress tumor progression in preclinical breast and colon model with independent regulatory T cells response
Source: Transl Oncol. 2022 Mar 24;20:101405. doi: 10.1016/j.tranon.2022.101405 (PMC8961218; doi:10.1016/j.tranon.2022.101405)
Supplement: Supplementary file 9 [file mmc9.docx]

**Anti-CTLA-4 and anti-PD-1 immunotherapies repress tumor progression in preclinical breast and colon model with independent regulatory T cells response**

Tristan Rupp^1,#^, Laurie Genest^1^, David Babin^1^, Christophe Legrand^1^, Marion Hunault^1^, Guillaume Froget^1^, Vincent Castagné^1^

Running title: Anti-CTLA-4 and anti-PD-1 repress tumor progression

# corresponding and lead author

[trupp@porsolt.com](mailto:trupp@porsolt.com), [rupptristan@hotmail.fr](mailto:rupptristan@hotmail.fr), Phone: 0033 (0)2 43 69 36 07

^1^ Porsolt SAS, French preclinical contract research organization (CRO), ZA de Glatigné, 53940 Le Genest-Saint-Isle, France

The authors declare no potential conflicts of interest

**List of abbreviations:**

EGFR: Epidermal Growth Factor

EMT: Epithelial-to-Mesenchymal Transition

ER: Estrogen Receptor

HER2: Human Epidermal Growth Factor Receptor 2

PR: Progesterone Receptor

RFS: Relapse Free Survival

SD: Standard Deviation

TILs: Tumor-infiltrating lymphocytes

TNBC: Triple Negative Breast Cancer

**Abstract**

The recent development of immunotherapy represents a significant breakthrough in cancer therapy. Several immunotherapies provide robust efficacy gains in a wide variety of cancers. However, in some patients the immune checkpoint blockade remains ineffective due to poor therapeutic response and tumor relapse. An improved understanding of the mechanisms underlying tumor-immune system interactions can improve clinical management of cancer. Here, we report preclinical data evaluating two murine antibodies corresponding to recent FDA-approved antibodies for human therapy, e.g. anti-CTLA-4 and anti-PD-1. We demonstrate in two mouse syngeneic grafting models of triple negative breast or colon cancer that the two antibodies display an efficient anticancer activity, which is enhanced by combination treatment in the breast cancer model. We also demonstrate that CTLA-4 targeting reduced metastasis formation in the colon cancer metastasis model. In addition, using cytometry-based multiplex analysis, we show that anti-CTLA-4 and anti-PD-1 affect the tumor immune microenvironment differently and in particular the tumor immune infiltration. This work demonstrated anti-cancer effect of CTLA-4 or PD-1 blockade on mouse colon and triple negative breast and on tumor-infiltrating immune cell subpopulations that could improve our knowledge and benefit the breast and colon cancer tumor research community.

**Keywords**

Checkpoint inhibitors

Immunotherapies

Immuno-oncology

Anti-PD-1

Anti-CTLA-4

Syngeneic preclinical models

Metastasis

**Introduction**

Cancer is a complex disease in which tumor cells interact with multiple stromal cells including immune cells within the so-called “tumor microenvironment” [1]. Recently, immunotherapies, in particular “checkpoint” inhibitors, demonstrate robust efficacy gains and durable responses in a wide variety of cancers, representing a significant therapeutic breakthrough [2–4]. Optimization of immune cell responses including enhancement of the cytotoxic T cell response has been described to promote tumor regression in vivo [5] and patient survival in clinical trials [2,4]. Response to immunotherapy relies on dynamic interactions between tumor cells and the tumor microenvironment, which may lead to an anti-cancer response from immune cells against pro-tumoral stromal cells or cancer cells. Targeting immune cells using “checkpoint” molecules, such as cytotoxic T-lymphocyte-associated antigen 4 (CTLA-4) [6] or programmed cell death protein 1 (PD-1) have demonstrated significant clinical responses and increased patient survival [7]. As an example, blockade of the CTLA-4 improves overall survival by more than 10 months for patients with metastatic melanoma [8,9]. The blockade of the PD-1/PD-L1/PD-L2 signaling axis leads to favorable clinical responses in advanced non–small-cell lung cancer, melanoma, prostate cancer, renal cancer, and colorectal cancer [10–14]. The combination of PD-1 and CTLA-4 blockade is also investigated, in particular at the preclinical level, and may represent a promising approach with even higher benefit for patients [15].

Despite huge clinical progress in the last past year, a detailed understanding of the mechanisms supporting anti-CTLA-4 and anti-PD-1 induced tumor immune rejection [16–18] is still lacking. Furthermore, a limited effect of the immunotherapies is observed in numerous of patients [19,20]. Indeed, tumor-associated events such as dynamic cell-cell interaction, modified microenvironment during tumor progression [19,21], or genetic alterations [18,22] can influence the therapeutic response to immunotherapies. An improved comprehension of the effects of checkpoint inhibitors may improve the clinical management of cancer, with ensuing long-term benefit for patients. Therapy with a combination of immunomodulatory agents is also emerging as an attractive option in the management of cancer [23]. Evaluation of the effects of mono- and combined-immunotherapy remains essential to anticipate the clinical benefit for patients, particularly in cases of tumor for which few effective therapeutic strategies are available.

In this study, we addressed the effects of monotherapy and combination therapy using anti-PD-1 and anti-CTLA-4 therapeutic antibodies in graft mouse tumor models. We demonstrate that the two antibodies affect the tumor immune microenvironment in different ways, even if they both display efficient anticancer activity. Anti-PD-1 and anti-CTLA-4 alone or in combination displayed different anti-cancer efficacy between the CT26 colorectal and the 4T1 triple negative breast cancer models. Interestingly, analysis of publically available patient databases with breast cancer, and in particular with triple negative patients, tends to suggest that high expression of interactors of PD-1, e.g. PD-L1 and PD-L2 and of CTLA-4, e.g. CD80 and CD86, generally expressed in the tumor microenvironment [24,25], are strongly correlated with the prognosis for patients. We also dissected the specific intratumoral immune response using flow cytometry and identified that anti-CTLA-4 acted by reducing regulatory T cells and increasing CD8+ T cells, although anti-PD-1 only increased intratumoral CD8+ T cells in both models. Finally, we also analyzed the response of immune-based therapies on metastasis formation and showed that anti-CTLA-4 reduced the metastatic burden in CT26 models without affecting metastasis in 4T1 models, whereas anti-PD-1 was devoid of effects in both models.

Altogether, these pre-clinical analyses suggest that the anti-tumor immune responses induced by CTLA-4 and PD-1 blockade may represent relevant therapeutic and potentially synergistic strategies, repressing tumor progression dependent of cancer indication.

**Material and Methods**

**Animals**

6 week-old female BALB/cJRj or C57BL/6JRj mice supplied by Janvier Labs were acclimated for at least 5 days before the experiments. The tumor cell implantation was performed on 7 to 8 week-old mice. Mice were housed up to 10 animals per cage in a biosafety level 1 laboratory. Nesting enrichment was provided (tube, cotton, and wood). Mice were maintained under artificial lighting (12 hours) between 7:00 and 19:00 in a controlled ambient temperature of 22 ± 2°C, and relative humidity between 30-70%. The number of mice per group included in each experiment is described in the legends of the corresponding figures.

**Cells and cell culture**

CT26.WT (CT26) colon carcinoma (CRL-2638™ from ATCC^®^) and 4T1 triple negative mouse breast carcinoma (CRL-2539™ from ATCC^®^) were cultured in vitro with RPMI 1640 (Gibco^®^, ATCC-formulated) supplemented with fetal bovine serum (FBS, Gibco^®^) at the final concentration of 10% and antibiotics (Penicillin 100U/mL - Streptomycin 100 µg/mL, Gibco^®^) and were grown in cell incubator at 37°C and 5% CO_2_. Prior to cell injection, cells at 70-90% confluence were split and cell viability was assessed using the automated cell counter Nucleocounter NC-200™ (Chemotec^®^). The cell suspension was prepared according to the viable cell count. All procedures were performed in aseptic conditions, under a laminar flow hood.

**Animal ethical consideration and limit points**

All methods, which were designed to minimize animal suffering and to ensure good quality of biological samples, are adapted from basic procedures commonly used in studies performed in rodents. Experiments were conducted in strict accordance with Council Directive No. 2010/63/UE of September 22^nd^ 2010 on the protection of animals used for scientific purposes, the French decree No. 2013-118 of February 1^st^ 2013 on the protection of animals for use and care of laboratory animals and with the recommendations of the Association for Assessment and Accreditation of Laboratory Animal Care (AAALAC). All experiments were also approved by the ethics committee for animal experimentation of Porsolt (Porsolt’s agreement n°C5301 031). Tumor volume and body weight of the animals were measured and recorded two to three times per week. Tumor volume exceeding 2000 mm^3^ and a weight loss greater than 20% relative to the initial weight of the animal for two consecutive measures, tumor necrosis including bleeding, ulceration, hypothermia (< 34°C), dyspnea, failure to eat and drink, loss of balance, and marked sedation were considered as limit points. When one of these conditions was met, mice were sacrificed by CO_2_ inhalation.

**Subcutaneous graft animal model**

5x10^5^ CT26 cells or 5x10^5^ 4T1 cells were injected subcutaneously into the right flank of the mice. The cells to be implanted were resuspended in sterile PBS and kept on ice. Mice were placed under anesthesia 2% isoflurane (Axience^®^, reference 152678) at 2 L/min on a warming pad and with eye lubricant during the procedure. The back of the mice was shaved and the area for injection was cleaned with Chlorhexidine (Antisept™, reference ANT015) before the injection of 100 µl of cell suspension using insulin syringe. Mice were identified by permanent tattoo. Finally, the mice were monitored (breathing) until they woke up.

Tumor volume was measured two to three times a week with a caliper. The tumor volume was calculated using the formula V = (*a^2^*b*)/2, where *b* is the longest axis and *a* is the perpendicular axis to *b*. The technician performing the measurement was not blinded with respect to the identity of the treatment received by the animals. Different physiological and behavioral parameters were monitored during the study including rectal temperature (hypothermia being defined as < 34°C), dyspnea, failure to eat and drink, loss of balance, and marked sedation.

Depending of model used, primary tumors and lungs were collected. Whole tissues were rapidly removed, rinsed in physiological saline, dried on absorbent paper, and weighed.

**Cytometry**

CT26 or 4T1 tumors were harvested 5 days after the last treatment and minced with scalpels. Up to 300 mg of the minced tissue was placed in a C-tube (130-095-823, Miltenyi Biotec™) containing 5 mL of PEB buffer (PBS, 0.5% bovine serum albumin, and2 mM EDTA), and then homogenized using the Miltenyi gentleMACS™. The sample was then transferred to a 50 mL conical tube through a 40 µm filter (352340, Becton Dickinson/Falcon™), and the filter was then rinsed with 5 mL PEB buffer. Immune cell population (CD45+) was enriched using CD45 MicroBeads and a MiniMACS™ Separator. Tumor infiltrating lymphocytes (TILs) were analyzed by flow cytometry analyses using the following antibody reagents: anti-CD8a-PerCP-eFluor-710 (clone 53–6.7; 46–0081, eBioscience™), anti-CD4-PerCP-Vio700 (clone REA1211, Miltenyi Biotec™), anti-CD25-PE (clone PC61.5; 12–0251, eBioscience™), anti-FoxP3-APC (clone FJK-16s; 17–5773, eBioscience™).

**Experimental metastasis model**

2x10^5^ CT26 cells or 2x10^5^ 4T1 cells were injected intravenously via the caudal vein using an insulin syringe. The cells to be implanted were resuspended in sterile PBS and kept on ice. Mice were placed under anesthesia 2% isoflurane (Axience^®^, reference 152678) at 2 L/min on a warming pad and with eye lubricant during the procedure. The tails of the mice were cleaned with Chlorhexidine (Antisept™, reference ANT015) before the injection. Tumor cells were injected within a volume of 100 µL. Mice were identified by permanent tattoo. Finally, the mice were monitored (breathing) until they woke up.

After 4 days, mice were randomized in the different groups based on their body weight and monitored for an additional 11 days. On Day 15, mice were sacrificed and 2 mL of 15% India Ink solution were intratracheally injected to stain the lungs, which were harvested, washed with distilled water and fixed overnight in Fekete’s solution. Tumor metastases are not stained and appear as white foci over a dark blue colored lung parenchyma. Left and right lungs were dried on absorbent paper and weighed. The left lung was pictured and metastasis surface area was measured by image analysis using executable software developed by Porsolt’s IT service in MATLAB.

**Treatments**

Anti-mouse CTLA-4 was purchased from BioXCell ^®^ (clone 9H10, reference BE0101, Rat IgG2b, κ and clone UC10-4F10-11, reference BE0032, Armenian Hamster IgG). Anti-mouse PD-1 was purchased from BioXCell^®^ (clone RMP1-14, reference BE0146, Rat IgG2a, κ).

Mice were randomized based on their tumor volume on the first day of treatment. Mice were treated with Anti-CTLA-4 at 100 µg per mouse (diluted in saline) or with Anti-PD-1 at 200 µg per mouse (diluted in saline). Drugs or vehicle were administrated through intraperitoneal route (i.p.), three to four time at day 3, 6, 9, 12, or 15.

**Clinical survival analysis**

The clinical data from breast cancer cohorts shown here are based on mixed publicly available data: E-MTAB-365*, GSE11121, GSE12093, GSE12276, GSE1456, GSE16391, GSE16446, GSE16716, GSE17705, GSE17907, GSE19615*, GSE20271, GSE2034, GSE20685, GSE20711, GSE21653*, GSE2603*, GSE26971, GSE2990, GSE31519*, GSE3494, GSE37946, GSE42568, GSE45255*, GSE4611, GSE5327, GSE6532, GSE7390, GSE9195. Overall survival (OS) was constructed using the Kaplan-Meier method with the online software ‘Kaplan-Meier Plotter’ (https://kmplot.com/analysis/) [26]. Probeset 1554519_at (CD80), 205685_at (CD86), 220049_s_at (PD-L2 or PDCD1LG2), 227458_at (PD-L1 or CD274) have been used. The log-rank test was used for comparison between low and high expressing groups. *Cohort including ER-, PR-, HER2- patients.

**Statistics**

Statistical analyses and graphical representations were done using GraphPad Prism (version 8.4.3). p values < 0.05 were considered as statistically significant (* p < 0.05; ** p < 0.01; *** p < 0.001; **** p < 0.0001). All data per group have been checked for normality using the D'Agostino-Pearson test. In case of normal distribution, a parametric test has been used, and in case of non-normal distribution, a non-parametric test has been used.

For lung metastasis and T cell tumor infiltration, data were analyzed using a one-way ANOVA or Kruskal-Wallis (group as factor). In case of a significant group effect, post-hoc Tukey’s or Dunn’s multiple comparison tests were done.

For tumor volume and body weight, data were analyzed using a two-way ANOVA (group and day as factors) with repeated measures at each day. In case of significant group and/or interaction effect, post-hoc Bonferroni’s multiple comparison tests (versus control, for each day) or Tukey’s multiple comparison tests (for each day) were done.

The cumulative survival distribution was constructed using the Kaplan-Meier method. Differences between survival curves were tested for significance with the log rank test.

**Results**

**Anti–CTLA-4 and –PD-1 repressed CT26 colon tumor progression in immunocompetent mouse model**

In order to identify the impact of mouse-based immunotherapies on CT26 tumors, we evaluated different schedules of treatment with anti-CTLA-4 and anti-PD-1 antibodies at respective doses of 100 and 200 µg per animal, starting from day 3, 6, or 9 and every 3 days with a total of three to four injections. We observed that the four treatment schedules with anti-CTLA-4 induced significant decrease of tumor growth starting from day 17 with the treatment at days 3, 6, 9, at days 3, 6, 9, 12, and at days 6, 9, 12 whereas starting from day 22 for treatment at days 9, 12, 15, 18 was not effective (**Figure S1A**). Regarding anti-PD-1 treatment, both regiments at days 6, 9, 12 or 9, 12, 15, 18 induced significant decrease of tumor growth at day 21 (**Figure S1B**). The treatment at day 6, 9, 12 demonstrated good anti-tumor response to the two antibodies and was used for the remainder of the study.

We also challenged different doses of anti-PD-1 and anti-CTLA-4 in order to identify optimal concentration of the treatment. Anti-CTLA-4 was used at 10, 50, and 100 µg and was administrated at days 6, 9, 12. We showed for all three doses a similar significant reduction of tumor volume starting from day 20 with the doses at 10 and 100 µg and starting from day 24 for the dose at 50 µg. Nevertheless and despite the absence significant difference between the three doses tested, the dose at 100 µg seems providing a better anti-cancer response in endpoint at day 29 (**Figure S2A**). Anti-PD-1 was used at 10, 100, and 200 µg and was administrated at days 6, 9, 12. We showed a significant reduction of tumor volume in a dose-response manner starting from day 20 with the dose at 200 µg, starting from day 24 for the dose at 100 µg, and starting from day 29 for the dose at 10 µg (**Figure S2B**). According to these results, the respective doses of 200 and 100 µg for anti-PD-1 and anti-CTLA-4 were used for the remainder of the study.

We also evaluated the effect of combination treatment of immunotherapies in comparison to monotherapies. We demonstrated again that both monotherapies with anti-CTLA-4 and anti-PD-1 reduced tumor progression in syngeneic CT26 colon cancer (**Figures 1A-E**). Interestingly, combined treatment significantly increased the response of anti-PD-1 but not of anti-CTLA-4 (**Figures 1A-E**). This effect of anti-CTL-A treatment seems masking the anti-PD-1 treatment. Interestingly, individual curves showed a higher proportion of mice without detectable tumors when receiving the combination (8/9) as compared to anti-CTLA-4 monotherapy (7/10) (**Figures 1C and 1E**). The Kaplan-Meier analysis demonstrated a global significant difference in term of survival between groups, which is significant when comparing the control group versus anti-CTLA-4 and combination groups (**Figures 1F**).

**Combination therapy of both immunotherapies enhanced anti-tumor activity in 4T1 breast tumor in immunocompetent mouse model**

We analyzed the effect of similar dosing and treatment schedule used with the CT26 model on a triple negative breast cancer model using 4T1 cells. We observed that both anti-PD-1 and anti-CTLA-4 weakly but significantly reduced tumor growth in a similar manner (**Figure 2A**). When compared to the CT26 model, immunotherapies are less efficient in the 4T1 model, which is described as less sensitive [27–32]. Combination therapy demonstrates significant anti-cancer efficacy compared to the control group but also compared to both monotherapies in the 4T1 model (**Figure 2A-E**); suggesting that low sensitivity to monotherapies might be overcome by combination therapy. No mice were found dead or required sacrifice for ethical limitation.

**Analysis of public clinical data: High expression of interactors of CTLA-4 and PD-1 are correlated with better prognostic value in breast cancer**

We analyzed a publically available dataset of patients with breast cancer and also with exclusive triple negative subtype. Patients were stratified between high and low expression using best cut-off value for dedicated interactors of CTLA-4, e.g. CD80 and CD86, but also interactors of PD-1, e.g. PD-L1 and PD-L2 [22]. We observed that high expression of the four cellular receptors expressed in the tumor microenvironment [22,24], correlated with longer relapse-free survival (RFS) of patients and interestingly, combined expression correlated better for better RFS, except when compared to PD-L1 alone (**Figure S3**). These data suggest that multiple targeting for immunotherapy might be of particular therapeutic interest, in particular when associated with our preclinical results showing enhanced anti-tumoral effect of combined therapy in the 4T1 graft model (**Figure 2A**). Altogether, these data suggest combination therapy might elicit a superior response compared to monotherapies, in particular for some forms of treatment-resistant cancers.

**Evaluation of tumor-infiltrating lymphocytes specific response to anti-CTLA-4 and -PD-1**

In order to evaluate the impact of the immunotherapies on the immune tumor microenvironment, we analyzed, by flow cytometry, the T cell population into the tumor, focusing on regulatory and CD8+ cytotoxic T cells among the CD45+ cells. Regulatory T cells was defined as CD45+/CD4+/CD25+/FoxP3+ event [33] whereas cytotoxic T cells was defined using CD45+/CD8+ event [34].

We observed that anti-CTLA-4 reduced the regulatory T cell population in the CT26 tumors. Conversely, anti-PD-1 did not affect the regulatory T cell population (**Figures 3A and 3B**), consistent with clinical observations [35]. We also demonstrated that anti-CTLA-4 significantly increased the CD8+ T cell population in the CT26 tumors whereas anti-PD-1 displayed a similar tendency (+99%, p < 0.05 and +71%, p=0.0748, respectively) (**Figures 3C and 3D**). We also calculated the ratio of CD8+ cytotoxic T cells / regulatory T cells which is a factor associated with better prognosis in patients with colon cancer [36] and we showed that anti-CTLA-4 increased the ratio, although anti-PD-1 did not (**Figure 3E**).

A similar analysis was performed on a small sample-size with 4T1 tumor models including this time the combination treatment. We also observed a significant decreased of regulatory T cells only with anti-CTLA-4 antibody and no change with anti-PD-1 (**Figure S4A**). Interestingly, the combination therapy did not improve and even cancelled the effect of anti-CTLA-4 (**Figure S4A**). As observed in the CT26 model, CD8+ T cells are enhanced by the immunotherapies in 4T1 tumors but combined treatments did not further improve this effect (**Figure S4B**). Finally, anti-CTLA-4 increased significantly the ratio of CD8+ T cells / regulatory T cells although anti-PD-1 did not (**Figure S4C**).

Despite efficient and similar anti-cancer activity (**Figures 1 and 2**), immunophenotyping analysis demonstrates different cellular mechanisms between CTLA-4 and PD-1 blocking strategies. Targeting CTLA-4 leads to a reduction of regulatory T cell population and to recruitment of CD8+ T cells, while targeting PD-1 induces only recruitment of CD8+ T cells (**Figures 3 and S4**).

**CTLA-4 targeting strategy reduced metastasis formation in CT26 colon experimental metastasis model**

We also investigated the effect of both immunotherapies on the intravenous-based experimental metastasis model [37]. This model allows analyzing the effect of substances on extravasation and lung parenchyma colonization processes [38]. Immunotherapies were tested in both 4T1 and CT26 metastasis models. We demonstrated that anti-CTLA-4, but not anti-PD-1, significantly reduced lung weight and metastasis surface in the lung in our CT26 colon cancer model (**Figures 4A-C**). Conversely, immunotherapies were devoid of effects in the 4T1 breast cancer model (**Figures 4D-F**).

**Immunotherapies were well tolerated in vivo**

We also investigated the effect of treatments on mouse body weight in order to identify potential drug obvious side effects. The presence of tumors was not associated with significant variations in body weight compared with non-inoculated mice. Both immunotherapies administered in monotherapy, or in combination, did not affect mouse body weight compared to the vehicle control in both CT26 and 4T1 subcutaneous models (**Figures 5A and 5B**). Moreover, no obvious change in mouse behavior was observed during the study. Altogether, these data suggest that immunotherapies are well tolerated in mice, even if additional analysis is still needed in order to confirm sign of toxicity.

**Discussion**

In this study, we demonstrated that CT-26 colon cancer and 4T1 triple negative breast cancer have different sensitivity to anti-PD-1 and anti-CTLA-4 therapy. CT-26 tumor highly responds to anti-CTLA-4 therapy whereas it is less sensitive to anti-PD-1. Combination therapy did not improve CT-26 tumor response compared to CTLA-4 alone; suggesting the absence of a cumulative effect, and therefore the main response is due to the exclusive action of CTLA-4 itself (**Figure 1**). Nevertheless, this phenomenon seems different between tumor models since Wei and collaborators demonstrated that combination therapy improved tumor reduction in MC38 colon cancer graft model [39]. Analysis of T-cell infiltration by flow cytometry demonstrated that only anti-CTLA-4 repressed regulatory T cells, which are well described promoters of tumor progression [33]. Anti-CTLA-4 also repressed lung metastasis burden in our experimental metastasis CT-26 model suggesting particular interest of CTLA-4 blockade for advanced colon cancer. Conversely, no effect on metastases was observed in 4T1 TNBC model. This absence of response might be related to the tumor immune microenvironment and T cells infiltration of 4T1 and CT26 tumors [40]. Indeed, 4T1 tumor has been considered as poorly responsive to immunotherapies [27–32], and aggressive metastatic cells might present more resistant phenotype as has been recently demonstrated for liver metastases [41]. Moreover, Anti-PD-1 did not induce similar anti-cancer effects and did not affect the population of regulatory T cells. Nevertheless, both anti-CTLA-4 and -PD-1 promoted cytotoxic T cells which seem to be involved in the reduction of tumor progression in this CT-26 model (even if only significant with anti-CTLA-4). Interestingly, the ratio of CD8+ T cells to regulatory T cells was enhanced after treatment with anti-CTLA-4. A high ratio of CD8+ or CD3+ on FOXP3 T cells has already been described as being correlated with a good prognosis in human colon cancer patients [36,42] and in breast cancer [34]. Some studies also observed that anti-CTLA-4 improves mice survival [43] or represses tumor growth with the CT26 model [44–46]. Moreover, CD8 and CD4 depletion inhibits the anti-CTLA-4 effect in CT26 subcutaneous models [47] suggesting a primordial role of T cell modulation in the tumor microenvironment as a mechanism of action behind the anti-cancer efficacy of anti-CTLA-4. Thus, CTLA-4 blockade through the recruitment of CD8+ cells within the tumor may promote better response as compared to the effect on regulatory T cells [48]. Moreover, recent finding demonstrated CTLA-4 blockade promotes regulatory T cells dysfunction through the modulation of glycolysis metabolism [49]. Taken together, monotherapy with anti-CTLA-4 might represent an alternative effective strategy against colon cancer to anti-PD-1. Moreover, combined therapy, demonstrating higher anti-cancer efficacy in another colorectal cancer model [39], may still represent a valuable strategy to promote patient survival, and might also serve in advanced colon cancer metastasis stage.

In the 4T1 triple negative breast cancer model, the effects of anti-PD-1 or anti-CTLA-4 monotherapies were relatively weak (**Figure 2**). 4T1 cells are described in the literature to be poorly sensitive to immunotherapies [27–32,50]. Interestingly, the combination therapy enhanced the anti-tumoral effect compared to monotherapy. Combination therapy might therefore represent a new avenue to treat resistance in some forms of aggressive cancers. Recent clinical trials for advanced metastatic breast cancer demonstrated the potential interest in immunotherapy, and in particular, the anti-PD-1-based strategy [51,52]. Current clinical studies are focusing on this type of therapeutic strategy that can hopefully transform non-responder patients into responders, or overcome acquired resistance to immunotherapy, thereby prolonging patient survival [51]. Moreover, combination treatment of immunotherapies has already demonstrated its potential against resistance to immunotherapies in other tumor indications [5,28,53]. We also showed by multiplex analysis that mRNA expression levels of compiled CD80/CD86, known receptors of CTLA-4 [25], and PD-L1/PD-L2, known receptors of PD-1 [24], demonstrated improved overall survival of patients with breast cancer, including all subtypes combined and with triple negative breast cancer. Moreover, combination treatment of immunotherapies has also been associated with better response to monotherapy in mouse model [50]. Taken together, our study might guide as basis for further translational research and suggest that dual blockade of PD-1 and CTLA-4 might enhance the therapeutic activity when compared to monotherapy in breast cancer, thereby unraveling potential benefit for patients.

In our manuscript, we demonstrated that immunotherapies have better efficiency in our CT26 colon cancer model in comparison to our 4T1 TNBC model. This work is in accordance with others preclinical studies [23,46,50]. The association between genetic variations and immunotherapy benefit has been identified in clinic. For example, the effects that tumor mutational burden on therapy response have been recently investigated as a predictive biomarker for response to immune checkpoint blockade [54]. Nevertheless, a recent study from the group of Shiaw-Yih Lin demonstrated that high tumor mutation burden, that has been proposed as a predictive biomarker, did not correlate with clinical benefits for patients treated with immune checkpoint blockade across solid cancer types using a large dataset from The Cancer Genome Atlas [55]. Conversely, both mismatch repair deficiency and microsatellite instability have been identified as effective predictors of immunotherapy response [56,57]. Colorectal adenocarcinoma demonstrated relatively higher mismatch repair deficiency as compared to breast cancer patients [56]. Moreover, high mismatch repair deficiency is associated with better therapeutic benefit at least with anti-PD-1 inhibition in phase II clinical studies [58,59]. High microsatellite instability is also observed in colorectal cancer patients with good response for immunotherapies [60]. However, CT26 cell line are described to present low microsatellite instability and mismatch repair proficiency [61,62] and 4T1 cell line are described to present mismatch repair proficiency [62], suggesting that the immunotherapy response is quite independent to such mutations. Recently, an Immunoscore has been proposed as a new approach for the classification of cancer in order to predict the response to T cell checkpoint inhibition. In this score, CD3+ and CD8+ T cell infiltration is evaluated within the tumor tissue defining ‘hot’, ‘altered’ and ‘cold’ immune tumors [40]. Classification of tumors based on their immune phenotype can partially explain clinical response to immunotherapies, with in general a higher T cell infiltration in the ‘hot’ phenotype which is associated with better clinical response [40,63]. High proportion of TNBC patients present high tumor-infiltrating lymphocytes (TILs) which is correlated with better overall survival [64]. The 4T1 and CT26 models are described to belong to tumors with differential immunoscore level. Indeed, the 4T1 model could be considered as a ‘cold’ phenotype with low response to immunotherapies [65], despite the CT26 could be considered as a ‘hot’ phenotype with good response to immunotherapies [66]. Altogether these data might participate to explain the immunotherapy response observed in this work and in clinical observations.

Interestingly, immunotherapies demonstrated a relatively good safety profile in our study, in accordance with other studies [14,67,68]. Indeed, Chalabi and collaborators demonstrated that neoadjuvant immunotherapy including PD-1 plus CTLA-4 blockade demonstrated promising therapeutic response and might become the standard of care for some subpopulation of patients [14]. These data are consistent with clinical reports of acceptable adverse events of immunotherapies in monotherapy or in combination [11,23,69,70] compared to other treatments, such as chemotherapy. Nevertheless, immune-related adverse events might emerge due to immune over-activation with such immunotherapies. These events are described to be particularly important in patients with pre-existing autoimmune conditions. Such patients should be monitored with caution but represents a minor risk counterbalanced by the potential advantage of the immunotherapy efficacy against tumor progression [71,72]. Convergent data on immunotherapy also tend to favor its clinical recommendation in new indications such as breast cancer. Immunotherapies kill cancer cells and improve patient survival by stimulating the immune response, such as cytotoxic T response [68,70]. Immunophenotyping analysis brings further insight into the effects of anti-CTLA-4 and anti-PD-1 immunotherapies, describing similar effects of both antibodies to induce an increase of the intratumoral cytotoxic T cell population, and the specific effect of anti-CTLA-4 in reducing the regulatory T cells. This information is valuable for a better understanding of the mechanisms of action of these new therapeutic strategies, and may lead to improved treatment efficiency or unravel tumor resistance.

Although immune checkpoint inhibitors may be effective, in clinic eventually relapse and tumor progression can be observed in some patients [68]. A similar phenomenon can be observed in our experiments with the heterogeneity of response within the treated groups. Indeed, despite the similar genetic background of mice or the use identical tumor cell line, some mice were sensitive whereas other mice could be considered as resistant with lack or lowered efficacy of checkpoint inhibitors (**Figure 1**). This strongly suggests that the local tumor microenvironment exerts a pressure on tumor cells which limits the effect of the therapy and might give rise to tumor evading [23,68]. Resistance mechanism to anti-PD-1 has been recently proposed to be associated with tumor-associated macrophages that could bypass the targeting of T cells [73] or with mutation in key signaling pathways such as JAK1/2 kinases inducing IFNγ lack of response [74,75].

In conclusion, we have confirmed an anti-cancer effect of both immunotherapies anti-CTLA-4 and anti-PD-1 in syngeneic tumor models with colorectal and triple negative breast cancer cells. This anti-cancer efficacy did not have any associated safety concerns. In addition, anti-CTLA-4 was able to repress the development of colorectal-derived lung metastasis in an experimental metastasis model with CT26 cells. Dual blockage by combination of anti-CTLA-4 and anti-PD-1 treatments demonstrated better efficiency in the 4T1 triple negative breast cancer model when compared to monotherapy. In association with clinical data, our study suggests that combination therapy might represent a new avenue for advanced breast cancer treatment. We also identified different cellular mechanisms of action in response to either anti-CTLA-4 or anti-PD-1, with a common increase of intratumoral cytotoxic T cells upon treatment, but a target-specific decrease of regulatory T cells upon anti-CTLA-4 only. We trust the present study and its associated findings will benefit the colorectal and breast cancer tumor research community.

**Author contribution**

**Tristan Rupp:** Conceptualization, Methodology, Formal analysis, Writing - Original Draft, Visualization, Supervision. **David Babin**: Investigation, Validation. **Laurie Genest**: Investigation, Validation. **Christophe Legrand**: Investigation. **Marion Hunault**: Investigation. **Guillaume Froget**: Supervision. **Vincent Castagné:** Methodology, Writing - Reviewing and Editing,

**Acknowledgements**

We thank David Pushett for reading the manuscript and providing helpful comments regarding English. We thank Jean-Philippe Guégan, Vincent Verbiest, Lucie Ekué, and Lucie Lecouflet for their technical and/or scientific feedback. We thank Claire Duquesnoy for illustrating the graphical abstract.

**Funding**

The work was fully supported by Porsolt SAS.

**Competing Interests**

The authors are or were all employees of Porsolt and declare no conflict of interest.

**Bibliography**

[1] Lorusso G, Rüegg C. The tumor microenvironment and its contribution to tumor evolution toward metastasis. Histochem Cell Biol 2008;130:1091–103. https://doi.org/10.1007/s00418-008-0530-8.

[2] Gong J, Chehrazi-Raffle A, Reddi S, Salgia R. Development of PD-1 and PD-L1 inhibitors as a form of cancer immunotherapy: a comprehensive review of registration trials and future considerations. J Immunother Cancer 2018;6. https://doi.org/10.1186/s40425-018-0316-z.

[3] Farkona S, Diamandis EP, Blasutig IM. Cancer immunotherapy: the beginning of the end of cancer? BMC Med 2016;14. https://doi.org/10.1186/s12916-016-0623-5.

[4] Sharma P, Allison JP. Immune Checkpoint Targeting in Cancer Therapy: Towards Combination Strategies with Curative Potential. Cell 2015;161:205–14. https://doi.org/10.1016/j.cell.2015.03.030.

[5] Fiegle E, Doleschel D, Koletnik S, Rix A, Weiskirchen R, Borkham-Kamphorst E, et al. Dual CTLA-4 and PD-L1 Blockade Inhibits Tumor Growth and Liver Metastasis in a Highly Aggressive Orthotopic Mouse Model of Colon Cancer. Neoplasia 2019;21:932–44. https://doi.org/10.1016/j.neo.2019.07.006.

[6] Leach DR, Krummel MF, Allison JP. Enhancement of antitumor immunity by CTLA-4 blockade. Science 1996;271:1734–6.

[7] Rowdo M, Paula F, Baron A, Urrutia M, Mordoh J. Immunotherapy in Cancer: A Combat between Tumors and the Immune System; You Win Some, You Lose Some. Front Immunol 2015;6. https://doi.org/10.3389/fimmu.2015.00127.

[8] Hodi FS, O’Day SJ, McDermott DF, Weber RW, Sosman JA, Haanen JB, et al. Improved Survival with Ipilimumab in Patients with Metastatic Melanoma. N Engl J Med 2010;363:711–23. https://doi.org/10.1056/NEJMoa1003466.

[9] Schadendorf D, Hodi FS, Robert C, Weber JS, Margolin K, Hamid O, et al. Pooled Analysis of Long-Term Survival Data From Phase II and Phase III Trials of Ipilimumab in Unresectable or Metastatic Melanoma. J Clin Oncol 2015;33:1889–94. https://doi.org/10.1200/JCO.2014.56.2736.

[10] Brahmer JR, Tykodi SS, Chow LQM, Hwu W-J, Topalian SL, Hwu P, et al. Safety and activity of anti-PD-L1 antibody in patients with advanced cancer. N Engl J Med 2012;366:2455–65. https://doi.org/10.1056/NEJMoa1200694.

[11] Garon EB, Rizvi NA, Hui R, Leighl N, Balmanoukian AS, Eder JP, et al. Pembrolizumab for the treatment of non-small-cell lung cancer. N Engl J Med 2015;372:2018–28. https://doi.org/10.1056/NEJMoa1501824.

[12] Gettinger SN, Horn L, Gandhi L, Spigel DR, Antonia SJ, Rizvi NA, et al. Overall Survival and Long-Term Safety of Nivolumab (Anti–Programmed Death 1 Antibody, BMS-936558, ONO-4538) in Patients With Previously Treated Advanced Non–Small-Cell Lung Cancer. J Clin Oncol 2015;33:2004–12. https://doi.org/10.1200/JCO.2014.58.3708.

[13] Topalian SL, Hodi FS, Brahmer JR, Gettinger SN, Smith DC, McDermott DF, et al. Safety, activity, and immune correlates of anti-PD-1 antibody in cancer. N Engl J Med 2012;366:2443–54. https://doi.org/10.1056/NEJMoa1200690.

[14] Chalabi M, Fanchi LF, Dijkstra KK, Van den Berg JG, Aalbers AG, Sikorska K, et al. Neoadjuvant immunotherapy leads to pathological responses in MMR-proficient and MMR-deficient early-stage colon cancers. Nat Med 2020;26:566–76. https://doi.org/10.1038/s41591-020-0805-8.

[15] Mahoney KM, Rennert PD, Freeman GJ. Combination cancer immunotherapy and new immunomodulatory targets. Nat Rev Drug Discov 2015;14:561–84. https://doi.org/10.1038/nrd4591.

[16] Vinay DS, Ryan EP, Pawelec G, Talib WH, Stagg J, Elkord E, et al. Immune evasion in cancer: Mechanistic basis and therapeutic strategies. Semin Cancer Biol 2015;35:S185–98. https://doi.org/10.1016/j.semcancer.2015.03.004.

[17] Spitzer MH, Carmi Y, Reticker-Flynn NE, Kwek SS, Madhireddy D, Martins MM, et al. Systemic Immunity Is Required for Effective Cancer Immunotherapy. Cell 2017;168:487-502.e15. https://doi.org/10.1016/j.cell.2016.12.022.

[18] Garrido F, Aptsiauri N, Doorduijn EM, Garcia Lora AM, van Hall T. The urgent need to recover MHC class I in cancers for effective immunotherapy. Curr Opin Immunol 2016;39:44–51. https://doi.org/10.1016/j.coi.2015.12.007.

[19] Sharma P, Hu-Lieskovan S, Wargo JA, Ribas A. Primary, Adaptive and Acquired Resistance to Cancer Immunotherapy. Cell 2017;168:707–23. https://doi.org/10.1016/j.cell.2017.01.017.

[20] O’Donnell JS, Teng MWL, Smyth MJ. Cancer immunoediting and resistance to T cell-based immunotherapy. Nat Rev Clin Oncol 2019;16:151–67. https://doi.org/10.1038/s41571-018-0142-8.

[21] Restifo NP, Smyth MJ, Snyder A. Acquired resistance to immunotherapy and future challenges. Nat Rev Cancer 2016;16:121–6. https://doi.org/10.1038/nrc.2016.2.

[22] Keenan TE, Burke KP, Van Allen EM. Genomic correlates of response to immune checkpoint blockade. Nat Med 2019;25:389–402. https://doi.org/10.1038/s41591-019-0382-x.

[23] Rotte A. Combination of CTLA-4 and PD-1 blockers for treatment of cancer. J Exp Clin Cancer Res 2019;38:255. https://doi.org/10.1186/s13046-019-1259-z.

[24] Guo L, Wei R, Lin Y, Kwok HF. Clinical and Recent Patents Applications of PD-1/PD-L1 Targeting Immunotherapy in Cancer Treatment—Current Progress, Strategy, and Future Perspective. Front Immunol 2020;11. https://doi.org/10.3389/fimmu.2020.01508.

[25] Walker LSK, Sansom DM. Confusing signals: Recent progress in CTLA-4 biology. Trends Immunol 2015;36:63–70. https://doi.org/10.1016/j.it.2014.12.001.

[26] Györffy B, Lanczky A, Eklund AC, Denkert C, Budczies J, Li Q, et al. An online survival analysis tool to rapidly assess the effect of 22,277 genes on breast cancer prognosis using microarray data of 1,809 patients. Breast Cancer Res Treat 2010;123:725–31. https://doi.org/10.1007/s10549-009-0674-9.

[27] Pilones KA, Kawashima N, Yang AM, Babb JS, Formenti SC, Demaria S. Invariant natural killer T cells regulate breast cancer response to radiation and CTLA-4 blockade. Clin Cancer Res Off J Am Assoc Cancer Res 2009;15:597–606. https://doi.org/10.1158/1078-0432.CCR-08-1277.

[28] Takeda K, Kojima Y, Uno T, Hayakawa Y, Teng MWL, Yoshizawa H, et al. Combination Therapy of Established Tumors by Antibodies Targeting Immune Activating and Suppressing Molecules. J Immunol 2010;184:5493–501. https://doi.org/10.4049/jimmunol.0903033.

[29] Grasselly C, Denis M, Bourguignon A, Talhi N, Mathe D, Tourette A, et al. The Antitumor Activity of Combinations of Cytotoxic Chemotherapy and Immune Checkpoint Inhibitors Is Model-Dependent. Front Immunol 2018;9. https://doi.org/10.3389/fimmu.2018.02100.

[30] Mosely SIS, Prime JE, Sainson RCA, Koopmann J-O, Wang DYQ, Greenawalt DM, et al. Rational Selection of Syngeneic Preclinical Tumor Models for Immunotherapeutic Drug Discovery. Cancer Immunol Res 2017;5:29–41. https://doi.org/10.1158/2326-6066.CIR-16-0114.

[31] Kim S-S, Harford JB, Moghe M, Rait A, Chang EH. Combination with SGT-53 overcomes tumor resistance to a checkpoint inhibitor. Oncoimmunology 2018;7. https://doi.org/10.1080/2162402X.2018.1484982.

[32] Demaria S, Kawashima N, Yang AM, Devitt ML, Babb JS, Allison JP, et al. Immune-Mediated Inhibition of Metastases after Treatment with Local Radiation and CTLA-4 Blockade in a Mouse Model of Breast Cancer. Clin Cancer Res 2005;11:728–34.

[33] Plitas G, Rudensky AY. Regulatory T Cells: Differentiation and Function. Cancer Immunol Res 2016;4:721–5. https://doi.org/10.1158/2326-6066.CIR-16-0193.

[34] Peng G-L, Li L, Guo Y-W, Yu P, Yin X-J, Wang S, et al. CD8+ cytotoxic and FoxP3+ regulatory T lymphocytes serve as prognostic factors in breast cancer. Am J Transl Res 2019;11:5039–53.

[35] Wang W, Lau R, Yu D, Zhu W, Korman A, Weber J. PD1 blockade reverses the suppression of melanoma antigen-specific CTL by CD4+CD25Hi regulatory T cells. Int Immunol 2009;21:1065–77. https://doi.org/10.1093/intimm/dxp072.

[36] Suzuki H, Chikazawa N, Tasaka T, Wada J, Yamasaki A, Kitaura Y, et al. Intratumoral CD8(+) T/FOXP3 (+) cell ratio is a predictive marker for survival in patients with colorectal cancer. Cancer Immunol Immunother CII 2010;59:653–61. https://doi.org/10.1007/s00262-009-0781-9.

[37] Cominetti MR, Altei WF, Selistre-de-Araujo HS. Metastasis inhibition in breast cancer by targeting cancer cell extravasation. Breast Cancer Targets Ther 2019;11:165–78. https://doi.org/10.2147/BCTT.S166725.

[38] Roarty K, Echeverria GV. Laboratory Models for Investigating Breast Cancer Therapy Resistance and Metastasis. Front Oncol 2021;11:645698. https://doi.org/10.3389/fonc.2021.645698.

[39] Wei SC, Anang N-AAS, Sharma R, Andrews MC, Reuben A, Levine JH, et al. Combination anti–CTLA-4 plus anti–PD-1 checkpoint blockade utilizes cellular mechanisms partially distinct from monotherapies. Proc Natl Acad Sci 2019;116:22699–709. https://doi.org/10.1073/pnas.1821218116.

[40] Galon J, Bruni D. Approaches to treat immune hot, altered and cold tumours with combination immunotherapies. Nat Rev Drug Discov 2019;18:197. https://doi.org/10.1038/s41573-018-0007-y.

[41] Yu J, Green MD, Li S, Sun Y, Journey SN, Choi JE, et al. Liver metastasis restrains immunotherapy efficacy via macrophage-mediated T cell elimination. Nat Med 2021;27:152. https://doi.org/10.1038/s41591-020-1131-x.

[42] Sinicrope FA, Rego RL, Ansell SM, Knutson KL, Foster NR, Sargent DJ. A low intraepithelial effector (CD3+):regulatory (FoxP3+) T-cell ratio predicts adverse outcome of human colon carcinoma. Gastroenterology 2009;137:1270–9. https://doi.org/10.1053/j.gastro.2009.06.053.

[43] Crittenden MR, Zebertavage L, Kramer G, Bambina S, Friedman D, Troesch V, et al. Tumor cure by radiation therapy and checkpoint inhibitors depends on pre-existing immunity. Sci Rep 2018;8. https://doi.org/10.1038/s41598-018-25482-w.

[44] Selby MJ, Engelhardt JJ, Quigley M, Henning KA, Chen T, Srinivasan M, et al. Anti-CTLA-4 Antibodies of IgG2a Isotype Enhance Antitumor Activity through Reduction of Intratumoral Regulatory T Cells. Cancer Immunol Res 2013;1:32–42. https://doi.org/10.1158/2326-6066.CIR-13-0013.

[45] Selby MJ, Engelhardt JJ, Johnston RJ, Lu L-S, Han M, Thudium K, et al. Preclinical Development of Ipilimumab and Nivolumab Combination Immunotherapy: Mouse Tumor Models, In Vitro Functional Studies, and Cynomolgus Macaque Toxicology. PLoS ONE 2016;11. https://doi.org/10.1371/journal.pone.0161779.

[46] Duraiswamy J, Kaluza KM, Freeman GJ, Coukos G. Dual Blockade of PD-1 and CTLA-4 Combined with Tumor Vaccine Effectively Restores T Cell Rejection Function in Tumors. Cancer Res 2013;73:3591–603. https://doi.org/10.1158/0008-5472.CAN-12-4100.

[47] Lewis KE, Selby MJ, Masters G, Valle J, Dito G, Curtis WR, et al. Interleukin-21 combined with PD-1 or CTLA-4 blockade enhances antitumor immunity in mouse tumor models. OncoImmunology 2018;7:e1377873. https://doi.org/10.1080/2162402X.2017.1377873.

[48] Du X, Tang F, Liu M, Su J, Zhang Y, Wu W, et al. A reappraisal of CTLA-4 checkpoint blockade in cancer immunotherapy. Cell Res 2018;28:416–32. https://doi.org/10.1038/s41422-018-0011-0.

[49] Zappasodi R, Serganova I, Cohen IJ, Maeda M, Shindo M, Senbabaoglu Y, et al. CTLA-4 blockade drives loss of Treg stability in glycolysis-low tumors. Nature 2021;591:652–8. https://doi.org/10.1038/s41586-021-03326-4.

[50] Kim K, Skora AD, Li Z, Liu Q, Tam AJ, Blosser RL, et al. Eradication of metastatic mouse cancers resistant to immune checkpoint blockade by suppression of myeloid-derived cells. Proc Natl Acad Sci U S A 2014;111:11774–9. https://doi.org/10.1073/pnas.1410626111.

[51] Emens LA. Breast Cancer Immunotherapy: Facts and Hopes. Clin Cancer Res Off J Am Assoc Cancer Res 2018;24:511–20. https://doi.org/10.1158/1078-0432.CCR-16-3001.

[52] Sugie T. Immunotherapy for metastatic breast cancer. Chin Clin Oncol 2018;7:7. https://doi.org/10.21037/cco.v0i0.19854.

[53] Kocak E, Lute K, Chang X, May KF, Exten KR, Zhang H, et al. Combination Therapy with Anti–CTL Antigen-4 and Anti-4-1BB Antibodies Enhances Cancer Immunity and Reduces Autoimmunity. Cancer Res 2006;66:7276–84. https://doi.org/10.1158/0008-5472.CAN-05-2128.

[54] Strickler JH, Hanks BA, Khasraw M. Tumor Mutational Burden as a Predictor of Immunotherapy Response: Is More Always Better? Clin Cancer Res Off J Am Assoc Cancer Res 2021;27:1236–41. https://doi.org/10.1158/1078-0432.CCR-20-3054.

[55] McGrail DJ, Pilié PG, Rashid NU, Voorwerk L, Slagter M, Kok M, et al. High tumor mutation burden fails to predict immune checkpoint blockade response across all cancer types. Ann Oncol 2021;32:661–72. https://doi.org/10.1016/j.annonc.2021.02.006.

[56] Le DT, Durham JN, Smith KN, Wang H, Bartlett BR, Aulakh LK, et al. Mismatch-repair deficiency predicts response of solid tumors to PD-1 blockade. Science 2017;357:409–13. https://doi.org/10.1126/science.aan6733.

[57] Zhao P, Li L, Jiang X, Li Q. Mismatch repair deficiency/microsatellite instability-high as a predictor for anti-PD-1/PD-L1 immunotherapy efficacy. J Hematol OncolJ Hematol Oncol 2019;12:54. https://doi.org/10.1186/s13045-019-0738-1.

[58] Overman MJ, McDermott R, Leach JL, Lonardi S, Lenz H-J, Morse MA, et al. Nivolumab in patients with metastatic DNA mismatch repair deficient/microsatellite instability–high colorectal cancer (CheckMate 142): results of an open-label, multicentre, phase 2 study. Lancet Oncol 2017;18:1182–91. https://doi.org/10.1016/S1470-2045(17)30422-9.

[59] Le DT, Uram JN, Wang H, Bartlett BR, Kemberling H, Eyring AD, et al. PD-1 Blockade in Tumors with Mismatch-Repair Deficiency. N Engl J Med 2015;372:2509–20. https://doi.org/10.1056/NEJMoa1500596.

[60] Overman MJ, Lonardi S, Wong KYM, Lenz H-J, Gelsomino F, Aglietta M, et al. Durable Clinical Benefit With Nivolumab Plus Ipilimumab in DNA Mismatch Repair-Deficient/Microsatellite Instability-High Metastatic Colorectal Cancer. J Clin Oncol Off J Am Soc Clin Oncol 2018;36:773–9. https://doi.org/10.1200/JCO.2017.76.9901.

[61] Wang Y, Wei B, Gao J, Cai X, Xu L, Zhong H, et al. Combination of Fruquintinib and Anti–PD-1 for the Treatment of Colorectal Cancer. J Immunol 2020;205:2905–15. https://doi.org/10.4049/jimmunol.2000463.

[62] Song W, Shen L, Wang Y, Liu Q, Goodwin TJ, Li J, et al. Synergistic and low adverse effect cancer immunotherapy by immunogenic chemotherapy and locally expressed PD-L1 trap. Nat Commun 2018;9:2237. https://doi.org/10.1038/s41467-018-04605-x.

[63] Vareki SM. High and low mutational burden tumors versus immunologically hot and cold tumors and response to immune checkpoint inhibitors. J Immunother Cancer 2018;6:157. https://doi.org/10.1186/s40425-018-0479-7.

[64] Althobiti M, Aleskandarany MA, Joseph C, Toss M, Mongan N, Diez-Rodriguez M, et al. Heterogeneity of tumour-infiltrating lymphocytes in breast cancer and its prognostic significance. Histopathology 2018;73:887–96. https://doi.org/10.1111/his.13695.

[65] Fabian KP, Padget MR, Fujii R, Schlom J, Hodge JW. Differential combination immunotherapy requirements for inflamed (warm) tumors versus T cell excluded (cool) tumors: engage, expand, enable, and evolve. J Immunother Cancer 2021;9:e001691. https://doi.org/10.1136/jitc-2020-001691.

[66] Sato Y, Fu Y, Liu H, Lee MY, Shaw MH. Tumor-immune profiling of CT-26 and Colon 26 syngeneic mouse models reveals mechanism of anti-PD-1 response. BMC Cancer 2021;21:1222. https://doi.org/10.1186/s12885-021-08974-3.

[67] Du X, Liu M, Su J, Zhang P, Tang F, Ye P, et al. Uncoupling therapeutic from immunotherapy-related adverse effects for safer and effective anti-CTLA-4 antibodies in CTLA4 humanized mice. Cell Res 2018;28:433–47. https://doi.org/10.1038/s41422-018-0012-z.

[68] Seidel JA, Otsuka A, Kabashima K. Anti-PD-1 and Anti-CTLA-4 Therapies in Cancer: Mechanisms of Action, Efficacy, and Limitations. Front Oncol 2018;8. https://doi.org/10.3389/fonc.2018.00086.

[69] Gu L, Khadaroo PA, Su H, Kong L, Chen L, Wang X, et al. The safety and tolerability of combined immune checkpoint inhibitors (anti-PD-1/PD-L1 plus anti-CTLA-4): a systematic review and meta-analysis. BMC Cancer 2019;19. https://doi.org/10.1186/s12885-019-5785-z.

[70] Wu K, Yi M, Qin S, Chu Q, Zheng X, Wu K. The efficacy and safety of combination of PD-1 and CTLA-4 inhibitors: a meta-analysis. Exp Hematol Oncol 2019;8. https://doi.org/10.1186/s40164-019-0150-0.

[71] Spiers L, Coupe N, Payne M. Toxicities associated with checkpoint inhibitors—an overview. Rheumatol Oxf Engl 2019;58:vii7–16. https://doi.org/10.1093/rheumatology/kez418.

[72] Haanen JB a. G, Carbonnel F, Robert C, Kerr KM, Peters S, Larkin J, et al. Management of toxicities from immunotherapy: ESMO Clinical Practice Guidelines for diagnosis, treatment and follow-up†. Ann Oncol 2017;28:iv119–42. https://doi.org/10.1093/annonc/mdx225.

[73] Arlauckas SP, Garris CS, Kohler RH, Kitaoka M, Cuccarese MF, Yang KS, et al. In vivo imaging reveals a tumor-associated macrophage mediated resistance pathway in anti-PD-1 therapy. Sci Transl Med 2017;9:eaal3604. https://doi.org/10.1126/scitranslmed.aal3604.

[74] Zaretsky JM, Garcia-Diaz A, Shin DS, Escuin-Ordinas H, Hugo W, Hu-Lieskovan S, et al. Mutations Associated with Acquired Resistance to PD-1 Blockade in Melanoma. N Engl J Med 2016;375:819–29. https://doi.org/10.1056/nejmoa1604958.

[75] Shin DS, Zaretsky JM, Escuin-Ordinas H, Garcia-Diaz A, Hu-Lieskovan S, Kalbasi A, et al. Primary resistance to PD-1 blockade mediated by JAK1/2 mutations. Cancer Discov 2017;7:188–201. https://doi.org/10.1158/2159-8290.CD-16-1223.

**Figure legends**

**Figure 1**: Effect of anti-CTLA-4 and anti-PD-1 immunotherapies in subcutaneous based syngeneic CT26 colorectal cancer model.

A. Impact of anti-CTLA-4 and -PD-1 immunotherapies in monotherapy or in combination on CT26 tumor growth. B-E Individual growth curve for each mouse treated with control (B), anti-CTLA-4 (C); anti-PD-1 (D), or combination (E).

Anti-CTLA-4 and anti-PD-1 were administered at days 6, 9, and 12 post-tumor cell inoculation at respective doses of 100 and 200 µg i.p. per animal. Statistical differences between the groups were determined using by two-ways ANOVA with repeated measures followed by Tukey multiple comparisons test (control vs. other groups: * p ≤ 0.05, ** p ≤ 0.01, *** p ≤ 0.001, **** p < 0.0001; anti-PD-1 vs. anti-CTLA-4 # p ≤ 0.001; anti-PD-1 vs. combination & p ≤ 0.0001). Pooled data represent mean ± SD. n = 9-10 mice per group.

**Figure 2**: Effect of anti-CTLA-4 and -PD-1 immunotherapies in subcutaneous based syngeneic 4T1 triple negative breast cancer model.

A. Impact of anti–CTLA-4 and –PD-1 immunotherapies in monotherapy or in combination on 4T1 tumor growth. B-E Individual growth curve for each mouse treated with control (B), anti-CTLA-4 (C); anti-PD-1 (D), or combination (E).

Anti-CTLA-4 and anti-PD-1 were administered at days 6, 9, and 12 post-tumor cell inoculation at respective doses of 100 and 200 µg i.p. per animal. Statistical differences between the groups were determined using by two-ways ANOVA with repeated measures followed by Tukey multiple comparisons test (control vs. other groups: * p ≤ 0.05, ** p ≤ 0.01, **** p < 0.0001; anti-CTLA-4 vs. combination # p ≤ 0.05; anti-PD-1 vs. combination & p ≤ 0.05). Pooled data represent mean ± SD. n = 10 mice per group.

**Figure 3:** Anti-CTLA-4 and anti-PD-1 involve independent cellular mechanisms related to their anti-cancer activity

A-B. FACS multicolor analysis (A) and quantification of regulatory T cells population (B) after tissue processing in the CT26 mouse colon cancer model. C, D. FACS multicolor analysis (C) and quantification of cytotoxic T cells population (D). E. Ratio of effector CD8+ T cells to T regs.

Anti-CTLA-4 and anti-PD-1 were administered with different regimens at respective doses of 100 and 200 µg i.p. per animal vehicle in saline (control). Statistical differences between the groups were determined using by one-way ANOVA test followed by Tukey’s multiple comparisons test (* p ≤ 0.05, **** p ≤ 0.0001). Data represent mean and SD. Combined data from different experiments. n = 14-23 mice per group.

**Figure 4:** Effect of anti-CTLA-4 and anti-PD-1 immunotherapies in syngeneic intravenous based experimental metastasis models

A-B. Representative pictures (A) and quantification of lung weight (B) after necropsy in the CT26 mouse colon cancer model. C, D. Representative pictures (C) and quantification of lung weight (D) after necropsy in the 4T1 mouse breast cancer model.

Anti-CTLA-4 and anti-PD-1 were administered at days 6, 9, and 12 post-cell inoculation at respective doses of 100 and 200 µg i.p. per animal or with vehicle (Control). Statistical differences between the groups were determined using Kruskal-Wallis test followed by Dunn’s multiple comparisons test (* p ≤ 0.05). Data represent mean and SD. N = 5-8 mice per group.

**Figure 5:** Impact of anti–CTLA-4 and anti-PD-1 immunotherapies in monotherapy or in combination on mice body weight

A-C. Impact of anti–CTLA-4 and –PD-1 immunotherapies in monotherapy or in combination on CT26 (A) and 4T1 (B) mouse body weight. Anti-CTLA-4 and anti-PD-1 have been administered at days 6, 9, and 12 at respective doses of 100 and 200 µg i.p. per animal. Statistical differences between the groups were determined using by two-ways ANOVA. Data represent mean and SD. n = 9-10 mice per group.

**Figure S1**: Treatment schedule-related effect of anti–CTLA-4 and anti-PD-1 immunotherapies in subcutaneous based syngeneic CT26 colorectal cancer model. Related to Figure 1.

A. Impact of anti–CTLA-4 immunotherapy on CT26 tumor growth. Anti-CTLA-4 was administered following different schedules at day 3, 6, and 9; 3, 6, 9, and 12; 6, 9, and 12; or 9, 12, 15, 18 post-tumor cell inoculation. B Impact of anti–PD-1 immunotherapy on CT26 tumor growth. Anti-CTLA-4 was administrated following different schedule at day 6, 9, and 12 or 9, 12, 15, 18 post-tumor cell inoculation.

Anti-CTLA-4 and anti-PD-1 were administered at respective doses of 100 and 200 µg i.p. per animal. Statistical differences between the groups were determined using by two-ways ANOVA with repeated measures followed by Bonferroni’s multiple comparisons test (versus control, * p ≤ 0.05, ** p ≤ 0.01, *** p ≤ 0.001, **** p < 0.0001). Data represent mean and SD. n = 5-10 mice per group (A) or n = 8-11 mice per group (B).

**Figure S2**: Dose-related effect of anti–CTLA-4 and anti-PD-1 immunotherapies in subcutaneous based syngeneic CT26 colorectal cancer model. Related to Figure 1.

A. Impact of different doses of anti–CTLA-4 immunotherapy on CT26 tumor growth. Anti-CTLA-4 was administered at 6, 9, and 12 post-tumor cell inoculation. B Impact of different doses of anti–PD-1 immunotherapy on CT26 tumor growth. Anti-PD-1 was administrated at 6, 9, and 12 post-tumor cell inoculation.

Anti-CTLA-4 has been administrated at 10, 50, and 100 µg i.p. per animal. Anti-PD-1 were administered at 10, 100, and 200 µg i.p. per animal. Control mice have been administrated with 200 µg of IgG antibody. Statistical differences between the groups were determined using by two-way ANOVA with repeated measures followed by Bonferroni’s multiple comparisons test (versus control, * p ≤ 0.05, ** p ≤ 0.01, *** p ≤ 0.001, **** p < 0.0001). Data represent mean and SD. n =10 mice per group.

**Figure S3**: Analysis of publicly available clinical data of CD80, CD86, PD-L1, and PD-L2 expression in breast cancer.

Expression of the CD80, CD86, PD-L1 (CD274), and PD-L2 (PDCD1LG2) are correlated with poor relapse-free survival (RFS) of breast cancers without subtype discrimination and triple negative breast cancer patients. Relapse-free survival analysis was done using Kaplan Meier curve of patients from patients with breast cancer. Patients were stratified in low or high-expressing groups according to the expression of the different genes using the best cut-off value. Analysis was performed either with all patients or with patients with a triple negative breast cancer subtype. The number of patients in each group is indicated below the graphs, p-values indicate the significance of survival difference between the groups of individuals by log-rank test.

**Figure S4:** Anti-CTLA-4 and anti-PD-1 exhibit independent cellular mechanisms leading to anti-cancer activity. Related to Figure 3.

A-C. Quantification by FACS multicolor analysis of regulatory T cells population (A) and cytotoxic T cells population (B) after tissue processing in the 4T1 triple negative breast cancer model. C. Ratio of effector CD8+ T cells to T regs.

Anti-CTLA-4 and anti-PD-1 were administered using different regimens in i.p. at respective doses of 100 and 200 µg per animal vehicle in saline (control). Data represent mean and SD. Statistical differences between the groups were determined using by Kruskal-Wallis test followed by Dunn’s multiple comparisons test (* p ≤ 0.05). Data represent mean and SD. Combined data from two different experiments. n = 5-6 mice per group.
