# Supplementary material for: Anti-CTLA-4 and anti-PD-1 immunotherapies repress tumor progression in preclinical breast and colon model with independent regulatory T cells response
Source: Transl Oncol. 2022 Mar 24;20:101405. doi: 10.1016/j.tranon.2022.101405 (PMC8961218; doi:10.1016/j.tranon.2022.101405)
Supplement: Supplementary file 8 [file mmc8.docx]

Dear Dr. Justin Durla Lathia,

First of all, we wish to thank the reviewers for the constructive comments about our manuscript.

We have made responses towards the comments as requested, and revised the manuscript accordingly. We resubmit the revised manuscript together with the point-to-point responses to the comments below. We note that some reviewers question the novelty of the manuscript. Nevertheless, we think that several points in our manuscript enhance knowledge in the field, including the evaluation of optimal immunotherapies schedule- and dose-response analysis, analysis of metastasis in a pure metastasis model, analysis of regulatory and cytotoxic T cell response in both CT26 colon and 4T1 breast cancer models within fresh tumors by cytometry, and clinical data about triple negative breast cancer patients.

Thus, we trust that this work is highly valuable for the scientific community and we hope that TRANON will positively consider the manuscript for publication.

Best regards,

Tristan Rupp, PhD

Reviewer Comments (if applicable):

Although your manuscript falls within the aim and scope of this journal, it is being declined due to lack of sufficient novelty. We receive a much larger number of papers than we are able to accept.

Reviewer #1: In this manuscript entitled "Anti-CTLA-4 and anti-PD-1 immunotherapies repress tumor progression through independent cellular mechanisms" authors provide data on the efficacy of the two major immunotherapies anti-CTLA4 and anti-PD1 (as monotherapies or in combination) in two commonly used syngeneic mouse models of cancer, namely CT26 and 4T1 ((colorectal and TNBC representative respectively). They also proved data some clinical data by analyzing the expressions of CD80/86 and PDL1/L2 in relation to RFS using publicly available data. From the data provided though I don't think that the title is sufficiently supported neither that "This work reveals a key target-specific effect of CTLA-4 or PD-1 blockade on mouse colon and triple negative breast tumor progression and on tumor-infiltrating immune cell subpopulations that could serve as a guide to improve therapeutic benefits for patients" as authors claim in the abstract.

Response to reviewer: We edited the title to be more specific. Moreover, we attenuated our comment in the abstract following the recommendation of the reviewer (lines 38-40).

To start with, regarding the cellular mechanisms of the two antibodies the only data that authors provide is the differences in the ratios of Tregs vs CD8s in the two syngeneic models which to me is very little to support "independent cellular mechanisms".

Response to reviewer: We edited the title of this work to be more specific.

Moreover there are several flaws regarding these data: authors did not perform this analysis in combination treated CT26 tumors while for the 4T1 model the data shown are very weak to support authors' statements. Authors show very few analyses in fig S4A and no statistics

Response to reviewer: We agree that the limited sample size analyzed in this study of 3 to 4 samples per group did not allow performing solid statistical analysis. Our initial goal was to give descriptive statistic for this model. We included now two additional samples and completed the analysis including statistics (Figure S4).

Also, If I am not wrong it seems to be a discrepancy in the number of tumors shown for ratio estimations (Fig 3E) as compared to CD8 and Tregs (Fig 3B and D. Is the population analysed in both cases the same?

Response to reviewer: The reviewer is right and the mistake included in the figure 3E has been corrected, the population analyzed was the same for the 3 graphs.

There are also several other flaws, discussed below, that weaken further the work:

In paragraph entitled "Anti-CTLA-4 and -PD-1 repressed CT26 colon tumor progression in immunocompetent mouse model" I am not sure I understand what the authors state in lines 219-221. Do they mean that the effect of the treatment is apparent starting from day 13? If this is what they mean is not accurate since the first time point with statistical significance is observed between days 15 and 18.

Response to reviewer: This was indeed a mistake, the significance of the difference started from day 17 and not 13. The text is revised accordingly (line 220).

Further down to the same paragraph (lines 236, 237) I am not sure whether this statement is supported by the data. What it seems to happen in this experiment is that, following this administration schedule, anti-CTLA4 is very potent masking the activity of the anti-PD1. So what is observed in the combination treatment is the effect of the anti-CTLA4. I don't think a synergism or additive effect could be claimed herein. To claim this authors should have used less potent dose/administration schedule for anti-CTLA4 and observe a better outcome in combination as compared to both monotherapies.

Response to reviewer: We do not claim in the manuscript regarding any synergistic or additive effect. This was even stated in the discussion section “Combination therapy did not improve CT-26 tumor response compared to CTLA-4 alone; suggesting the absence of a cumulative effect, and therefore the main response is due to the exclusive action of CTLA-4 itself (**Figure 1**).“ (lines 319-321)

Additionally to support their statement about more cured mice in combination vs anti-CTLA4 monotherapy the authors must provide Kaplan Meier survival curves.

Response to reviewer: we attenuated our comment and only mentioned that the proportion of mice with palpable tumor is more limited in the combination vs. anti-CTLA-4 groups and added the survival analysis for the CT26 model (Figure 1F). No mice died in the 4T1 model and thus no graph was included. A sentence describing this result was included in the manuscript (lines 256-257).

Regarding the 4T1 model to my understanding authors applied for 4T1 tumors the same dose/administration schedule as for the CT26. I wonder if this is the best schedule since 4T1 are known to be a very aggressive tumor (which is also obvious comparing the growth curves of the two models in this study). Could the outcome be better using higher doses or more frequent administrations?

Response to reviewer: these two hypotheses concerning the administration schedule fall beyond the scope of the present manuscript, but it might be interesting to investigate more in a further study.

In lines 258, 259 authors mention the expression of the four antigens in tumor or stromal cells of the tumor microenvironment. How these two populations were distinguished? Which population authors show in the corresponding graphs? How about colorectal cancer that is represented by the CT26 model?

Response to reviewer: We understand that the mention of “stromal cells” might be confusing and should not be included here. Indeed, these antigens are expressed by cancer cells but could also be expressed by antigen presenting cells such as dendritic cells (Peng et al., Nature Communications volume 11, 4835, 2020). Even if such cells could be present in the tumor microenvironment it is not our purpose with this analysis for which we did not discriminate expression-level, since the analysis is based on transcriptomic datasets from human tumor tissues. We removed this mention in the text (line 264).

Regarding the comment on colorectal cancer, these antigens are also expressed in colorectal cancer patient tissues (Lau et al., Nature Communications volume 8, 14572, 2017; Shi et al., PLoS One. 2013; 8(10): e76012; Wang et al., Cancer Cell International volume 21, 58, 2021). We did not investigate on the link between expression-level and prognosis because we think that this complex question falls out of the scope of the present study.

Regarding the metastasis experiment authors again don't perform any analysis in the combination treated CT26 and there is no effect whatsoever on 4T1 mets. Moreover authors don't provide any data on the cellular mechanisms of the two antibodies regarding their anti-metastatic potential which would be of great interest since they observe this important difference at CT26 tumors. So the information provided by this experiment is very limited and partial.

Response to reviewer: We understand the limitation of these data regarding the mechanism behind this observation, but to our knowledge it is an important finding since the effect of these immunotherapies on pure metastatic model were not well investigated. We hope studies by other authors will confirm our observations.

Also the number of lungs shown in Fig 4C are not in agreement with the data provided in Fig 4B regarding the anti-CTLA4 treatment. Are the data for these two analyses coming from the same or different experiments?

Response to reviewer: We gratefully thank the reviewer for identifying this mistake generated due to an error that has been corrected in the new version of the figure.

Overall this study needs to be greatly strengthened by providing more solid and conclusive data.

Response to reviewer: Thanks to the reviewer’s comments, we improved the manuscript and we hope that the current version provides additional information.

Reviewer #2: In the manuscript by Rupp et al. titled, "Anti-CTLA-4 and anti-PD-1 immunotherapies repress tumor progression through independent cellular mechanisms", the authors share their original research regarding the application of combination immunotherapies to treat triple negative breast cancer and colon cancer in mice models grafted with mouse cancer cells. The work sheds additional light on the fact that each cancer type and model used to study it has molecular nuances that shift the outcomes and efficacy of different therapies. Unfortunately, there was no accompanying molecular characterization that helps the community understand why CT-26 allografts were responsive to anti-CTLA-4 and not anti-PD-1 therapy. In addition to lacking mechanistic insights to truly be characterized as an advancement in scientific knowledge, there are some major and minor concerns that should be addressed.

Response to reviewer: We hope that the improvement included in the new version of our manuscript based on the reviewer’s comment, provide now enough additional information.

Major Corrections:

1. Line 128-129, The parameters the authors chose to measure are good signs of toxicity, however they do not adequately measure the most common side effects of these therapies - autoimmune toxicities. I recommend that the authors note this limitation but also cite the fact that these therapies are FDA approved and have shown very good tolerance in humans to support the claim that toxicity is of minimal concern.

Response to reviewer: We added a paragraph emphasizing the risk of autoimmune toxicities in the discussion (lines 400-406). Nevertheless, this risk may be considered as quite rare in the general population and is more related to patient with pre-existing autoimmune diseases (Spiers et al. Rheumatology (Oxford). 2019 Dec; 58(Suppl 7)).

2 Lines 320-321, the authors state that the difference in response between the two cancer types may be due to CD3+ infiltrates, could this experiment be performed and the data added to the paper? Response to reviewer: Despite the interest of such analysis, we are not considering to perform CD3 counting that could be considered out of the scope of the study. Indeed, our present work focuses on the efficacy of treatments rather than on their mechanism of action. Nevertheless, we are so far preparing another manuscript including more complete analysis of the tumor immune microenvironment using several methodologies in which CD3+ cells are included.

3. The discussion would greatly benefit from the authors noting the effects that tumor mutational burden can have on therapy response. This may also help them to determine why the colorectal and breast cancer lines responded differently.

Response to reviewer: We thank the reviewer for its interesting comment, and a paragraph has been added in the discussion in order to identify potential findings explaining the differential response observed including particularly recent findings on immune classification of tumor and tumor mutation burden (lines 367-395).

4. Anti-PD-1 was used at 10, 100, and 200 µg per injection and showed significant reduction of tumor volume in a dose-response manner starting from day 20 with the doses at 200 µg, starting from day 24 for the dose at 100 µg, and starting from day 29 for the dose at 10 µg. Allowing tumors different times to develop can introduce a bias into how the drugs perform.

Response to reviewer:

There is a misunderstanding, and we apologize for being unclear in the preceding version of the manuscript. All mice were randomized at day 6 when treatment with the three doses was started, and thus all tumors were allowed to grow during the same times before treatment. We only noticed that the significant difference appeared at different times. The text of the manuscript has been clarified (lines 227-228 and 231-232).

Minor Corrections:

5. Materiel and Methods. Material is spelt wrong.

Response to reviewer: Edited in the text

6. 'Prior to cell injection, cells as 70-90% confluence were split and cell viability was assessed using the automated cell counter Nucleocounter NC-200™ (Chemotec® 113).' Change as to at.

Response to reviewer: Edited in the text

7. 'All methods, which are designed to minimize animal suffering and to ensure good quality of biological 119 samples, are adapted from basic procedures commonly used in studies performed in rodents.' Change are to were.

Response to reviewer: Edited in the text

8. 'The sample was then transferred to a 50 mL conical tube through a 40 mM filter (352340, Becton Dickinson/Falcon™).' Change 40 mM to 40 mm.

Response to reviewer: Edited in the text

Reviewer #3: Rupp and colleagues describe the effects of anti-PD-1, anti-CTLA4, and the combination of anti-PD-1 plus anti-CTLA4 in the CT26 and 4T1 mouse models of cancer. Observations included tumor growth, Treg and CD8 immune cell infiltration, metastasis, and moues body weight. This paper is well written and easy to ready. However, the novelty of this work is very low as anti-PD1 and anti-CTLA4 therapy have been extensively studied as monotherapies and in combination in these models and in human patients.

Response to reviewer: although other articles also focused on some findings investigated in our manuscript, there are several points that improve knowledge in the field, including immunotherapies schedule- and dose-response analysis, analysis of metastasis in a pure metastasis model, analysis of regulatory and cytotoxic T cell response in both CT26 colon and 4T1 breast cancer models, and clinical data about triple negative breast cancer patients.

Thus, we trust that this work is highly valuable for the scientific community.

Major Points:

1. In figure 3, the authors claim that anti-CTLA4 functions through depletion of Tregs while anti-PD1 does not. It has been demonstrated many times that the antitumor efficacy of anti-CTLA in mouse models is heavily dependent on the depletion of Tregs. Furthermore, clinical analyses have shown that anti-CTLA4 does not deplete Foxp3 Tregs in human cancers (Sharma, Subudhi … Allison, Ribas, Sharma, Clin Cancer Res. 2019), so this mechanism of action seen in mice is unlikely to impact human patients. Authors should elaborate on this and describe if anti-CTLA4 therapy is different in the CT26 model. Data should also be reported for the anti-PD1/CTLA4 combination group.

Response to reviewer: We thank the reviewer for this interesting comment. As mentioned, Sharma's group demonstrated that the infiltration of CD8+ and CD4+ T cells are increased without depleting intra-tumoral Tregs in melanoma patient tissue. However, these results are in contradiction with another study in patients with metastatic melanoma treated with ipilimumab, which demonstrates a strong reduction in the number of intra-tumoral Tregs post-treatment in responder patients (Romano et al., Proc Natl Acad Sci U S A. 2015 May 12; 112(19): 6140–6145). The discrepancy may result from the analysis of biopsies performed late post-treatment. This depletion is probably no longer detectable 5 to 8 weeks post-treatment due to the recruitment of unaltered peripheral Tregs. In addition, given the low percentage of responder patients, it would have been interesting to segregate on the response to treatment since this decrease should only be observed in responders.

To our knowledge, no similar analysis was described for colon or breast cancer and the results from preclinical models remain of interest.

2. In figure 3, authors claim that both anti-CTLA4 and anti-PD1 function through the upregulation of cytotoxic T cells in the TME. Only CD45 and CD8 were used in flow cytometry gating. To show that these T cells are truly cytotoxic and not exhausted, an activation maker should be utilized such as Granzyme, TNF, IL-12, other cytokine production, etc. Are any of these T cells tumor antigen specific? For all flow cytometry, cell populations were reported as a percentage of CD45+ cells. Counts of cells per tumor weight would be more beneficial to support author claims. Data should also be reported for the anti-PD1/CTLA4 combination group.

Response to reviewer: We did not elaborate on the active status of CD8+ cells in this study, we limited the work to the analysis of CD8+ cells within the tumor microenvironment. We think that could be considered as cytotoxic but not necessarily active ones as also recognized in the literature (Peng et al., Am J Transl Res 2019;11:5039–53). Interestingly, CD8+ infiltrating cells is used in a ratio with FOXP3+ cell as predictive marker for survival at least in colorectal cancer (Suzuki et al., Cancer Immunology, Immunotherapy, 12 Nov 2009, 59(5):653-661) favoring the interest of this marker. Nevertheless, in order to be more specific we edited the text to include CD8+ cells as replacement of cytotoxic T cells. These modifications do not affect our conclusion.

3. In figure 3, extensive flow cytometry analysis for most of the main TME immune cell infiltrates should be performed and reported including CD4, NK, macrophage, MDSC, DC.

Response to reviewer: In this study, we decided to focus on the analysis of FoxP3+ and CD8+ cells and their modulation upon the different treatments. We are so far working on another manuscript including more complete analysis of the tumor immune microenvironment using several methodologies. Some of the cell types suggested by the reviewer are included in this ongoing study.

4. In figure 4, authors show the effect of anti-CTLA4 and anti-PD1 on control of iv tail vein metastasis for CT26 and 4T1. No experiment data explaining the mechanism of this is provided. Additionally, mammary fat pad s.c. implanted 4T1 is spontaneously metastatic to the lungs. This methodology would be more accurate for the 4T1 model. Data should also be reported for the anti-PD1/CTLA4 combination group.

Response to reviewer: We agree with the reviewer that the orthotopic 4T1 model is interesting since it provides the drug response on the primary tumor and on metastases. In our study, we nevertheless focused on a pure metastasis model limiting to study extravasation and parenchyma colonization processes. Thus, our primary question was not to see if immunotherapies can prevent metastasis formation, as could be achieved with orthotopic model in which metastases appear after establishment of primary tumors, but to see if immunotherapies can treat metastasis already formed. That’s why we preferred to use a pure metastasis model ([Kähkönen](https://www.ncbi.nlm.nih.gov/pubmed/?term=K%26%23x000e4%3Bhk%26%23x000f6%3Bnen%20TE%5BAuthor%5D&cauthor=true&cauthor_uid=32570871) et al., Cancers (Basel). 2020 Jun; 12(6): 1615).
